# Human Breast Milk Composition and Function in Human Health: From Nutritional Components to Microbiome and MicroRNAs

**DOI:** 10.3390/nu13093094

**Published:** 2021-09-02

**Authors:** Dae Yong Yi, Su Yeong Kim

**Affiliations:** 1 Department of Pediatrics, Chung-Ang University College of Medicine, Seoul 06974, Korea; meltemp2@hanmail.net; 2 Department of Pediatrics, Chung-Ang University Hospital, Seoul 06973, Korea

**Keywords:** human milk, microbiota, human health

## Abstract

Human breast milk (HBM) is not only an indispensable source of nutrients for early human growth and development, supplying components that support infant growth and development, but also contains various essential immunologic components with anti-infectious activities and critical roles in the formation of immunity. It is also known that HBM contains its own unique microbiome, including beneficial, commensal, and potentially probiotic bacteria, that can contribute to infant gut colonization. In addition, HBM-derived extracellular vesicles, exosomes, and microRNA are attracting increasing interest for their potential to transfer to the infant and their role in infant development. In this article, we examine some of the various constituents in HBM and review the evidence supporting their associated health effects and their potential applications in human health.

## 1. Introduction

Human breast milk (HBM) contains various components with critical roles in supporting early human growth and development [1,2]. Macronutrients are representative HBM components. The factors affecting these macronutrients and the associated benefits of HBM macronutrients for infant health have been well-studied over the past decades and continue to attract intense research attention. Changes in HBM according to the lactation stage, race, diet of the nursing mother, region-specific environmental chemicals, storage and treatment have been demonstrated [3,4,5]. It is well-recognized that breastfeeding prevents and lowers the prevalence of diseases [6,7]. Extensive data demonstrate that breastfed children have a lower incidence of many acute or chronic diseases, such as otitis media, acute diarrhea, lower respiratory tract infections, sudden infant death syndrome, inflammatory bowel disease, juvenile leukemia, diabetes, obesity, asthma, and atopic dermatitis [7,8]. It is known that exclusive breastfeeding for the first 6 months of age lowers mortality against infectious diseases by 88% and lowers the possibility of death compared to partial breastfeeding as a dose dependent effect [9].

**Figure 1:**
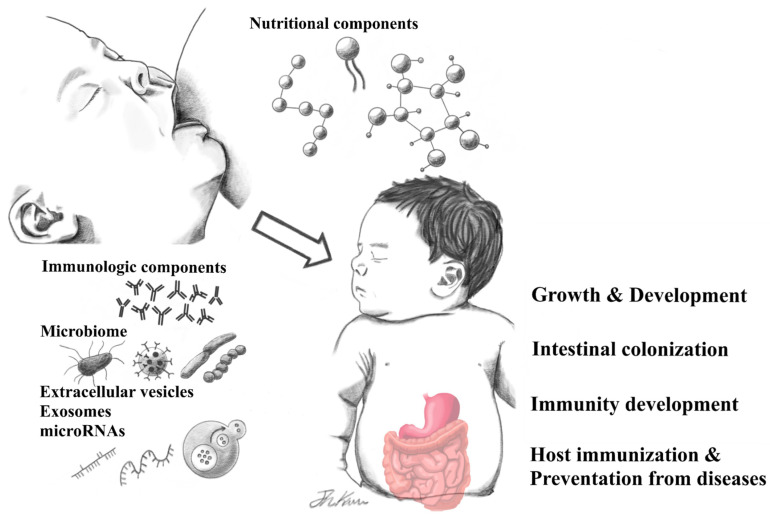
Human breast milk-derived components and their health effects.

The increasing sophistication and integration of advanced analytical technologies, such as next-generation sequencing, has enabled unprecedented exploration of HBM and expanded research on its various health effects. However, HBM is extremely complex and highly variable. New evidence has been presented in recent years, highlighting the limited data that currently exists on this most crucial aspect of infant nutrition [1,2]. In this review, we examine some of the various components in HBM and survey the literature on their health effects and potential human health applications (Figure 1).

## 2. Nutritional Components of HBM and Associated Human Health Benefits

Excluding the water, which comprises about 87–88%, the most basic components in HBM are macronutrients: carbohydrates, proteins, and fats [1,2]. These macronutrients provide essential nutritional support for infant growth and development, supplying 65~70 kcal of energy per 100 mL. HBM components change dynamically according to lactation periods and nursing sessions within a single feeding and become suitable for various needs according to the state of lactation or the child’s growth [3]. Carbohydrate, which comprises about 7% (60–70 g/L) of HBM, accounts for 40% of the total calorie reserve. Lactose is the main carbohydrate in HBM. It is decomposed and absorbed in the form of monosaccharides (glucose and galactose) by an enzyme called lactase-phlorizin hydrolase (lactase). Lactose is present in a higher concentration in HBM than in the milk of other species, reflecting the high energy demands of the human brain, showing positive associations with infant weight gain [9,10,11]. Insufficient lactase may cause lactose malabsorption, but it is relatively rare in exclusively breastfed infants [3]. It may occur secondary to acute diseases involving the small intestine, such as enteritis, and may affect infant growth when accompanied by chronic diseases, such as intestinal failure. Lactose is also the major contributor to the osmolality of HBM. From colostrum to transitional milk and mature milk, the average content of lactose increases slightly. By maintaining a fairly constant concentration in mature milk, HBM can maintain a constant osmotic pressure [3,12]. In addition, lactose aids the absorption and attachment of bioactive components, such as oligosaccharides and minerals and calcium [3].

HBM protein, composed of a mixture of whey, caseins, and various peptides, provides crucial amino acids indispensable for infant growth and development, as well as bioactive proteins and peptides essential for many functions. Caseins are present as micelles, which exists in the form of curd or clot in the stomach and are the primary source of calcium and phosphate in HBM, and whey protein exists in solution. Some proteins, such as α-lactalbumin, β-casein, folate-binding protein, haptocorrin, bile salt-stimulated lipase, amylase, α-1 antitrypsin, and lactoferrin, play an auxiliary role for digestion and utilization of various other nutrients [9,13]. Protein accounts for about 1% (8–10 g/L) of the HBM, presenting a relatively high content of 14–16 g/L at the beginning of the infant’s birth, but decreasing to 8–10 g/L at 3–4 months. After 6 months, it decreases to 7–8 g/L [2].

Fat, which accounts for almost 50% of the nutritional supply of the infant, is the second most prevalent macromolecule in HBM and is the most important for infant growth and development of the central nervous system [2]. LCPUFAs delivered through HBM affect retina and brain cortex development of infants [9]. Sphingomyelins, which affect the myelinisation of the central nervous system, play an important role in neurobehavioral effects, especially in underweight births [14]. In addition, DHA begins to accumulate in nervous tissues before birth, which is easily incorporated into infant brains during the first 2 years when the central nervous system differentiates and grows rapidly. In general, HBM contains 3.5–4.5% fat, of which 95–98% is in the form of triglycerides [3]. The fat content in HBM is affected by differences in dietary habits, maternal diet, weight change during pregnancy, and breast fullness including last feeding time. Immediately after birth, colostrum, the first HBM produced after birth, contains 15–20 g/L fat, but it gradually increases with lactation, reaching 40 g/L in mature milk. The fatty acid profile of HBM does not change during a single lactation even though the fat content increases 2–3 times from the foremilk to hindmilk [15]. Most of the fatty acids in HBM are C10-C18, with a small amount of unsaturated long-chain fatty acids. The long-chain polyunsaturated fatty acids (LCPUFAs) and their precursors are provided to breast-feeding infants through HBM in amounts that depend largely on the maternal diet. Docosahexaenoic acid (C22:6*n*-3), eicosapentaenoic acid (C20:5*n*-3), and the total *n*-3 LCPUFAs of HBM affect infant adiposity, and the human milk n-6/n-3 ratio has a positive relationship with infant body fat percentage [10,16]. The short-chain fatty acids in HBM are important sources of calories for infant growth and play an important role in gastrointestinal maturation [9,17].

## 3. Immunologic Components of HBM and Associated Human Health Benefits

In addition to nutritional biomolecules, HBM has many non-nutritional bioactive components that profoundly impact infant survival and health. Colostrum is particularly rich in immunologic components with anti-inflammatory and anti-infectious effects and known roles in regulating early intestinal colonization and immune development. Representative immunologic proteins include α-lactalbumin, lactoferrin, lysozyme, and secretory immunoglobulin A (sIgA), which are all whey proteins [1,18]. The predominance of whey proteins (90:10) in colostrum highlights their essential role in immune protection at birth [19].

sIgA constitutes about 80–90% of the total immunoglobulins in HBM. Approximately 0.5 to 1.0 g/day of this protein is ingested by infants fed exclusively with HBM. Only about 10% of this is absorbed by the intestine and transferred to the bloodstream, reflecting the local immune function of sIgA, that is, to deliver acquired immunity from mothers through the enteromammary pathway to support local immunity in the newborn [20,21]. sIgA molecules also impact the binding of pathogens, such as *Escherichia coli*, *Vibrio cholerae*, *Campylobacter*, *Haemophilus influenzae*, rotavirus, cytomegalovirus, and *Candida albicans*, preventing their adherence to the intestinal mucosal surface [3,19]. One study has suggested that sIgA may influence the pathogenesis of necrotizing enterocolitis (NEC) [22].

sIgA and lactoferrin account for about 26% of the protein in HBM, corresponding to a lactoferrin concentration of about 1 g/L in mature milk and 7 g/L in colostrum. Lactoferrin is an antimicrobial compound with a high affinity for iron. It exerts a bacteriostatic activity against iron-requiring pathogens and exhibits bactericidal activity against some pathogens [19]. In addition, lactoferrin affects the production and expression of various cytokines that affect the immune system.

Lysozyme inhibits the propagation of pathogenic bacteria (especially Gram-negative bacteria) by its synergetic action with lactoferrin [23,24]. Several studies have suggested that lysozyme has the potential to protect infants from intestinal inflammatory insult associated with NEC [24]. Another predominant whey protein in HBM known to play various roles in infants is α-lactalbumin. α-Lactalbumin is essential for lactose biosynthesis and facilitates the absorption of trace elements and minerals, such as calcium and zinc [3,19]. Furthermore, α-lactalbumin binds to oleic acid to form a complex known as human α-lactalbumin made lethal to tumor cells (HAMLET) that induces tumor apoptosis and activates ion fluxes to different cellular compartments and is expected to be applied clinically to treat various oncologic diseases [25,26]. The effects of intramural administration of HAMLET on brain glioblastoma, skin papilloma and bladder cancer were confirmed. In particular, its clinical potential was confirmed through a double blinded Phase I/II interventional clinical trial for bladder cancer [27]. In previous studies, bactericidal activity of HAMLET and effectiveness of combination treatment with antimicrobial agents showed the potential for the future use against select species of bacteria such as pathogenic streptococcal infections caused by resistant organisms [28].

Additionally, HBM contains cytokines, such as tumor necrosis factor-α, interleukin (IL)-1β, IL-6, IL-8, IL-10, interferon-γ, and transforming growth factor-β, which provide immunomodulation and passive protection, reducing the likelihood of infection [19,29].

Another important component in the formation of neonate immunity is the human milk oligosaccharides (HMOs). HMOs are the second most abundant carbohydrates after lactose and the third most abundant component overall in HBM [30]. Establishing an immune system against various infections immediately after birth is essential because the gastrointestinal tract of infants is sterile. In addition to direct ingestion of bioactive components through HBM, the formation of gut immunity through the acquisition of intestinal colonization is also important in boosting the developing infant immune system and providing protection against pathogens. HMOs are resistant to hydrolysis in the neonatal small bowel, so they are not absorbed and reach the large bowel mostly intact [31]. These HMOs serve as prebiotics and metabolic substrates, supporting the growth of beneficial gut bacteria and host defense [32]. HMOs are assimilated by infant gut-associated bifidobacteria, which are involved in the production of short-chain fatty acids, an important energy source for enterocytes [32]. The formation of a bifidobacteria-rich microbiota is considered important for long-term gut health. Furthermore, HMOs protect infants from pathogens, such as *Streptococcus pneumonia*, *E. coli*, Group B *Streptococcus*, and *Campylobacter*, by inhibiting their adhesion to the intestinal mucosa [11]. Additionally, preclinical animal studies and human studies support the preventive effects of HMOs against NEC in preterm infants [24,33]. It is noteworthy that human milk lipid components (especially medium-chain monoglycerides) have also been shown to have protective effects against several pathogens, such as Group B *Streptococcus* [34].

## 4. The HBM-Derived Microbiome

Historically, HBM was thought to be sterile [35]. The presence of bacteria in HBM was considered contamination or infection, so early studies of bacteria in HBM mainly dealt with mastitis or vertical infections through HBM [35,36,37]. The paradigm shifted after the early 2000s when several studies demonstrated the existence of commensal bacteria in HBM [38,39,40,41,42,43,44]. Early culture-based studies reported rapidly growing culturable Gram-positive bacteria, such as *Staphylococcus*, *Streptococcus*, *Corynebacterium*, and *Propionibacterium*. However, one study using culture techniques isolated lactic acid bacteria (LAB) [39]. None of the LAB isolated from breast skin shared identical DNA profiles with LAB isolated from HBM and mammary areola, suggesting that breastfeeding could be an important source of LAB for the infant’s gut [39]. Subsequent pioneering culture-dependent studies confirmed commensal bacteria in HBM [38,41,44]. The development of culture-independent analysis techniques, especially the emergence of high-throughput next-generation sequencing techniques, has broadened the horizons of research on the HBM microbiome. It is now widely accepted that abundant and diverse microorganisms exist in HBM [42,45,46] and that these microorganisms play an important role in infant gut colonization [47].

**Figure 2:**
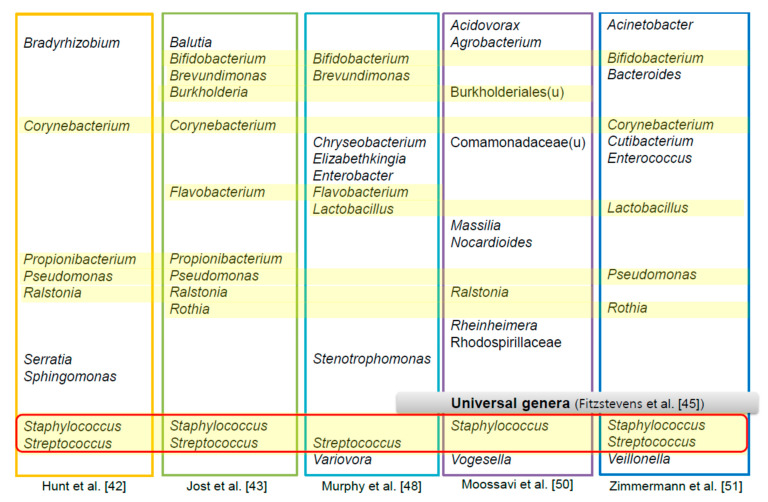
Core genera of human breast milk microbiome in several studies [42,43,45,48,50,51].

Beyond proving the presence of microbial communities in HBM, interest is focusing on identifying the representative genera and the role that the HBM microbiome plays in maternal and infant health (Figure 2). The first next-generation sequencing study to investigate the HBM microbiome suggested that there is a “core” gut bacterial microbiota (bacteriome) present in all samples and that it accounts for half of the microbial population [42]. This core bacteriome included nine genera: *Staphylococcus*, *Streptococcus*, *Serratia*, *Pseudomonas*, *Corynebacterium*, *Ralstonia*, *Propionibacterium*, *Sphingomonas*, and *Bradyrhizobium* [42]. However, these results were inconsistent with subsequent studies [43,48,49,50]. One study identified a core bacteriome of 12 genera: *Staphylococcus*, *Streptococcus*, *Bifidobacterium*, *Balutia*, *Brevundimonas*, *Corynebacterium*, *Flavobacterium*, *Propionibacterium*, *Pseudomonas*, *Ralstonia*, *Rothia*, and *Burkholderia* [43]. In another study, *Streptococcus*, *Elizabethkingia*, *Variovorax*, *Bifidobacterium*, *Flavobacterium*, *Lactobacillus*, *Stenotrophomonas*, *Brevundimonas*, *Chryseobacterium,* and *Enterobacter* comprised the core 12 genera [48]. In a large study that analyzed the HBM of 393 mothers in the Canadian Healthy Infant Longitudinal Development (CHILD) cohort, 100% of the samples contained 12 core genera [50]. The five most abundant belonged to unclassified Burkholderiales, *Staphylococcus*, *Ralstonia*, unclassified Comamonadaceae, and *Acidovorax*. The others were *Massilia*, *Rheinheimera*, *Agrobacterium*, unclassified Rhodospirillaceae, *Vogesella*, *Nocardioides*, and unclassified Burkholderiales [50].

A systematic review on healthy mother’s milk using only culture-independent techniques suggested that *Staphylococcus* and *Streptococcus* are universally predominant genera in HBM [45]. A recent review of 44 studies on bacterial communities in HBM reported that a core microbiome of 7–9 bacterial genera was mainly found [51]. The most frequently found genera were *Staphylococcus*, *Streptococcus*, *Lactobacillus*, *Pseudomonas*, *Bifidobacterium*, *Corynebacterium*, *Enterococcus*, *Acinetobacter*, *Rothia*, *Cutibacterium*, *Veillonella*, and *Bacteroides*. Another systematic review on the microbiome of human breast tissue and milk analyzed 242 studies (192 culture-based studies, 14 studies of 16S rRNA gene amplicon sequencing, and three studies of shotgun metagenomics) [46]. A total of 820 species, mainly composed of Proteobacteria and Firmicutes, highlighted the rich diversity of the HBM microbiome. The most frequently detected species were *Staphylococcus aureus*, *Staphylococcus epidermidis*, *Streptococcus agalactiae*, *Cutibacterium acnes*, *Enterococcus faecalis*, *Bifidobacterium breve*, *E. coli*, *Streptococcus sanguinis*, *Lactobacillus gasseri*, and *Salmonella enterica*.

There can be several reasons for the discrepancies in the findings mentioned above. First, individual, regional, and environmental factors may affect the microbial ecosystem of HBM. There is some evidence that parity, delivery mode, gestational age, biological sex, parity, intrapartum antibiotics, lactation stage, diet, body mass index, composition of breast milk, HIV infection, ethnicity, geographic location, and collection/feeding method influence the composition of the HBM microbiome [50,51]. However, the number of samples covered in many studies has been small, further contributing to the controversy [46,51]. Second, HBM sampling and processing are not standardized for each study. There are different processes, such as whether to use an aseptic technique for collecting HBM, express HBM by hand or pump, discard the first few drops or not, and the time lapse between collection and examination of the HBM [45,46,51]. Third, the experimental techniques used across studies are not the same, and the difference between laboratories is not calibrated. In particular, it is important to reduce errors in results due to contamination or technical errors and conduct unbiased analysis. Establishing a systematic and standardized research method in investigating the HBM microbiome will be a fundamental challenge, but is important to the advancement of our understanding of HBM microbiome.

The origin of bacterial populations in HBM has yet to be determined. The widely accepted hypotheses are contamination from the surface skin and infant’s oral cavity and translocation through the enteromammary pathway. Early studies that suggested microorganisms in HBM were contaminants from the mother’s skin when breastfeeding were based on similarities between skin microorganisms and milk microorganisms, such as *Staphylococcus* and *Corynebacterium* [52,53]. One study showing the retrograde flow of milk from the infants’ mouth back into the mammary ducts during suckling suggested that skin and oral microorganisms affect the formation of the HBM microbiome [54]. The enteromammary pathway means that maternal gut bacteria penetrate the epithelium and travel through an endogenous route to the mammary glands. Evidence supporting this theory is that the dendritic cells can penetrate the gut epithelium by opening the tight junctions, allowing the transfer of commensal bacteria from the gut lumen [55]. The presence of an enteromammary circulation of IgA-producing cells also supports this theory [56]. Meanwhile, studies comparing the microbiome composition of skin and HBM reported marked differences [42,57]. Several studies also observed that anaerobic bacteria, such as *Bifidobacterium*, *Bacteroides*, *Parabacteroides*, and *Clostridium*, which are absent from adult skin, are shared between HBM and infant feces [39,41,48,58].

## 5. Relationship between the HBM Microbiome and Human Health

It is now widely accepted that the HBM microbiome contributes to the gut colonization of the infant. Breastfeeding allows the HBM microbiome to enter the infant’s gut and serve as an inoculation. Studies have shown that some strains of species, such as *Lactobacillus* and *Bifidobacterium*, are shared in mother’s HBM and infant’s feces, supporting that HBM contributes to the vertical transmission of commensal bacteria [39,47,53,59,60]. However, there is still a lack of understanding of the exact mechanism. It is also necessary to identify which bacteria delivered to the infant through the HBM microbiome play a major role in immune development and beneficial symbiosis.

As part of this concept, an early study documented the anti-staphylococcal activity of *Lactobacillus rhamnosus* and *Lactobacillus crispatus* isolated from HBM [38]. *Lactobacillus* spp. have been shown to exert inhibitory activity against pathogens, such as *Shigella* spp., *Salmonella* spp., and *E. coli,* by preventing intestinal adhesion [44,61,62]. Some evidence supports the assumption that the relative abundance of *Bifidobacterium* is related to disease or immune development. A systematic review of 248 studies compared the composition of HBM in healthy mothers with those of infectious patients [46]. It showed that some bacterial species were found only in infected patients, while others existed only in healthy controls. In particular, *Bifidobacterium* and *Lactobacillus* were linked to the absence of infection. The gut microbiome of infant feces usually has a high abundance of *Bifidobacterium*, especially in exclusively breastfed infants [63,64].

In addition to the microbiome, HBM contains extracellular vesicles (EVs) of bacterial origin [65,66]. EVs are generally classified as apoptotic bodies, microvesicles, and exosomes. Bacteria-derived EVs of HBM are thought to be involved in the formation of infant gut colonization and immunity and to act as receptors for bioactive molecules in host cells [66,67,68]. We previously analyzed bacteria-derived EVs and found a high abundance of *Lactobacillus* in bacterial EV samples, as well as a high correlation between *Bifidobacterium* and bacterial EVs. These findings suggested the presence of key bacteria with metabolic activity in HBM.

Traditionally, *Lactobacillus* and *Bifidobacterium* have a long history as the most common and safely used probiotics [69]. However, through a deeper understanding of the HBM microbiome, it is expected that new probiotic strains will be isolated that are better transferred to the gut and show higher efficacy. Furthermore, factors that ultimately contribute to symbiosis in infants should be established, as such knowledge may allow manipulation of the infant gut microbiome for improved health when required.

It is well-known that HBM bacteriome dysbiosis, characterized by the rapid growth of pathogenic bacteria (*Staphylococcus* and/or *Streptococcus* and *Corynebacterium*) and depletion of commensal bacteria (*Lactococcus* and *Lactobacillus*), causes mastitis [70,71,72,73]. In addition, there is a growing interest in finding links between breast cancer and the microbial ecology in the mammary environment. A study showed differences in the bacterial composition of breast tissue between healthy women and those with breast cancer [74]. *Escherichia coli* and *S. epidermidis*, isolated from breast cancer patients, were shown to induce DNA double-stranded breaks. It is expected that a deeper knowledge of the ecology of the HBM microbiome will open more ways to predict and prevent various maternal diseases.

## 6. HBM-Derived Exosomes and microRNA in Relation to Human Health and Disease

Exosomes identified in surrounding body fluids, such as blood, urine, cerebrospinal fluid, saliva, and amniotic fluid, exhibit various health effects by transporting bioactive molecules, such as DNA, mRNA, microRNA (miRNA), lipids, and proteins [75,76,77]. Milk-derived exosomes protect intestinal epithelial cells from oxidative stress by regulating cell proliferation and inflammation and exert various physiological and therapeutic functions in immunomodulation and cancer [66]. Although one study could not confirm the direct antitumor effects of HBM exosomes, it indicated that HBM exosomes promoted the proliferation of normal colon epithelial cells without influencing the growth of colonic cancer cells [78]. The relationship between the incidence and treatment of various cancers, including breast cancer, lung, colon pancreatic, prostate, and ovarian cancers, is being studied [66].

HBM is highly enriched in miRNAs, short, non-coding RNAs of 18 to 25 nucleotides in length, involved in the development, differentiation, proliferation, metabolism, and death of cells and tissues [79,80]. Each miRNA has been shown to regulate the expression of multiple genes. Some of the tens of thousands of known species are reported to be involved in cancer generation, and aberrantly expressed miRNAs in certain types of cancer are emerging as possible biomarkers for diagnosis, treatment, and prognosis of various cancers [81,82]. Approximately 1400 miRNAs are delivered through HBM, with several implicated in the maturation of the immature immune system [83]. HBM miRNAs synthesized in mammary glands are delivered to infants by lactation, absorbed by epithelial cells of the intestinal wall, and then delivered to various organs through the bloodstream [83,84]. Diverse functions have been described for miRNAs isolated from HBM, including immune system development, such as immune response control and viral defense, and influencing the differentiation and maintenance of tissue identity, such as adipose tissue development and lung development [83]. Some miRNAs are key regulators of milk lipid metabolism. With roles in fatty acid oxidation, cholesterol homeostasis, and as critical regulators of lipogenesis, miRNAs may be useful in the treatment of diseases, such as atherosclerosis and dyslipidemia [85,86]. Additionally, certain miRNAs have been linked to growth control, apoptosis, epigenetic modifications, developmental programming, stem cell differentiation, and increase or decrease cancer risk by targeting genes involved in cancer [83]. In animal models, many breast milk miRNAs were associated with synaptic plasticity, cognitive capacity, and neurologic disorders [87]. Some evidence suggests an association between inflammatory bowel disease and NEC through the influence of miRNAs on intestinal maturation and inflammation [88]. Further research on HBM-derived miRNA and clinical approaches in humans can be expected to provide future therapeutic applications in disease such as cancer, infection, and neonate diseases.

## 7. Conclusions

HBM is a highly complex system of various bioactive components. It is the most suitable source of nutrients for infants and is indispensable in the formation of early immunity. Each component positively affects human health, such as early human immunity and disease prevention, independently and directly, and also exerts health effects via various interactions. Many bioactive components in HBM have not yet been identified, and knowledge of their role remains at the animal model or research level. Nevertheless, various bioactive components of HBM are expected to be useful in the diagnosis and treatment of diseases. In this article, we examined some of the various constituents in HBM and reviewed the evidence supporting their associated health effects and their potential applications in human health.
